# Three-dimensional printing of the retina

**DOI:** 10.1097/ICU.0000000000000252

**Published:** 2016-04-07

**Authors:** Barbara Lorber, Wen-Kai Hsiao, Keith R. Martin

**Affiliations:** a Centre for Brain Repair, University of Cambridge, Cambridge, UK; b Paracelsus Medical University, Salzburg, Austria; c Research Center Pharmaceutical Engineering GmbH, Graz, Austria; d Cambridge NIHR Biomedical Research Centre; e Eye Department, Addenbrooke's Hospital; f Wellcome Trust – Medical Research Council Cambridge Stem Cell Institute, Cambridge, UK; ∗ Barbara Lorber, Wen-Kai Hsiao, and Keith R. Martin contributed equally to the writing of this article.

**Keywords:** three-dimensional printing, functional retina, retinal cells

## Abstract

**Purpose of review:**

Biological three-dimensional printing has received a lot of media attention over recent years with advances made in printing cellular structures, including skin and heart tissue for transplantation. Although limitations exist in creating functioning organs with this method, the hope has been raised that creating a functional retina to cure blindness is within reach. The present review provides an update on the advances made toward this goal.

**Recent findings:**

It has recently been shown that two types of retinal cells, retinal ganglion cells and glial cells, can be successfully printed using a piezoelectric inkjet printer. Importantly, the cells remained viable and did not change certain phenotypic features as a result of the printing process. In addition, recent advances in the creation of complex and viable three-dimensional cellular structures have been made.

**Summary:**

Some first promising steps toward the creation of a functional retina have been taken. It now needs to be investigated whether recent findings can be extended to other cells of the retina, including those derived from human tissue, and if a complex and viable retinal structure can be created through three-dimensional printing.

## INTRODUCTION

Interest in three-dimensional printing has been phenomenal over the past few years. This technology, originally developed to produce engineering prototypes in plastics and metals, has been adapted to create biological structures. Although cell printing has been researched extensively [1–4], promising recent advances in the construction of tissue for transplantation, including skin tissue [5], heart tissue [6], bone [7], and tracheal structures [8], combined with extensive media coverage, has created the impression that any structure of the body, including a functional retina, can be custom made within the blink of an eye. There are, however, numerous challenges in the creation of complex organs of the body and functioning neuronal tissue that need to be addressed first.

We provide a review of recent progress toward constructing complex structures of the central nervous system like the retina. We also suggest steps that will be required if creation of functional retinal tissue to cure blindness is to be achieved. 

**Box 1:**
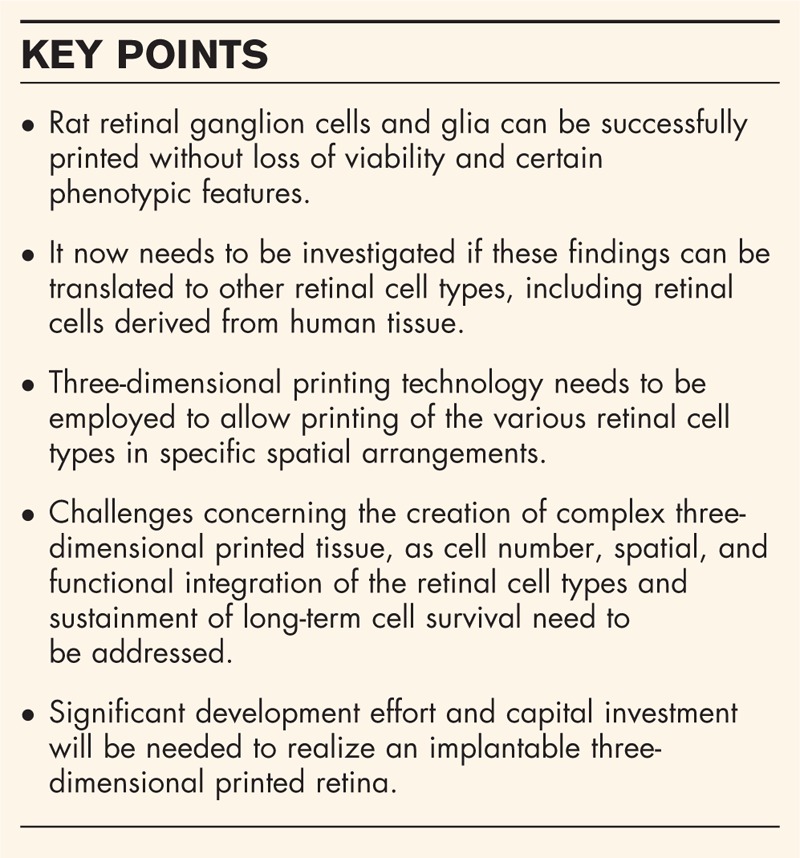
no caption available

## NEURONAL CELL PRINTING

Inkjet printing, a technology that is fundamental to many three-dimensional printing processes, has been successfully used to print several types of mammalian neuronal cells and to create cellular structures. Typical home and office desktop inkjet printers are based on the thermal inkjet principle in which the impulse to eject a liquid drop is provided by the expansion and collapse of a thermally-generated vapour bubble behind a nozzle. Such printers have been successfully modified to print cells, including muscle and stem cells, as well as embryonic neuronal cell types, including motor, hippocampal, and cortical neurons [1,2,4].

Until recently it had not been tested whether neuronal cells of the adult mammalian central nervous system, which have less survival and regeneration potential compared with their embryonic counterparts [9], would withstand the printing process.

## RETINAL CELL PRINTING

**FIGURE 1:**
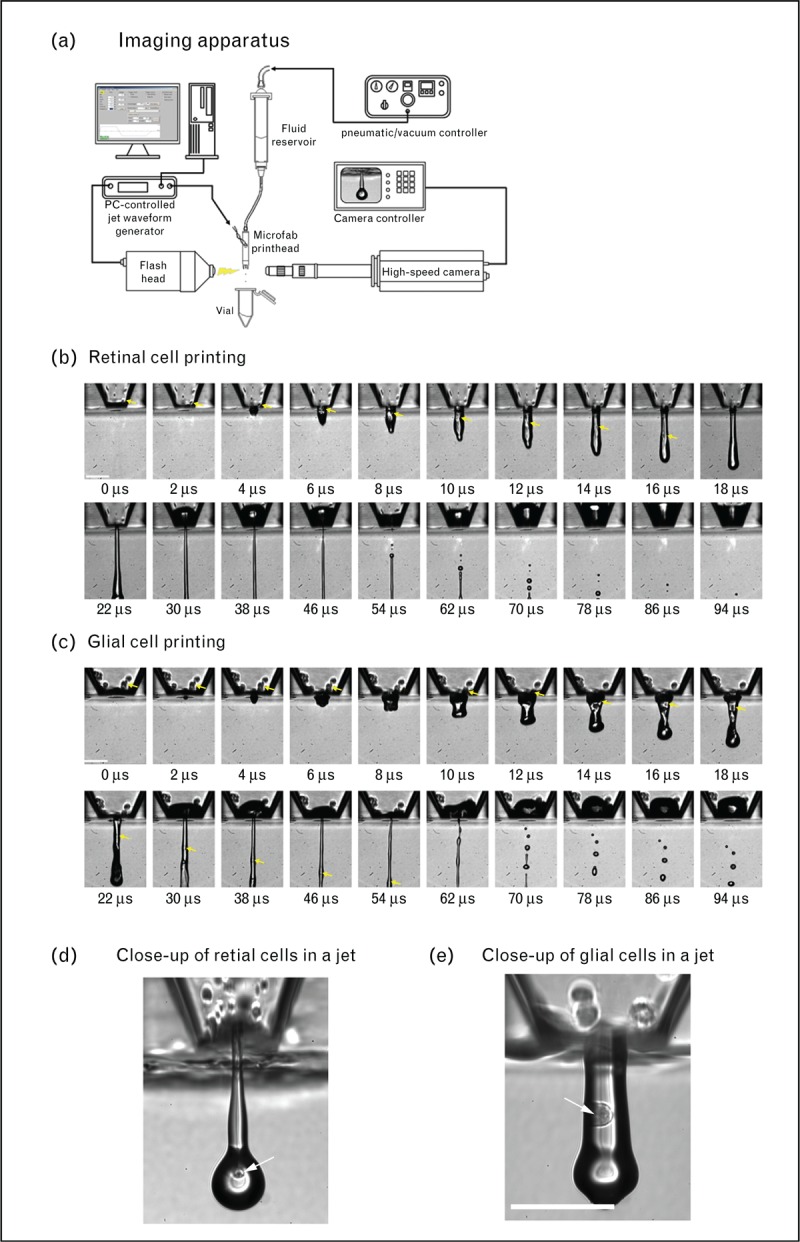
(a) Schematic of an inkjet printing and imaging apparatus that was used to print purified retinal glial and dissociated retinal cells. Image sequences of (b) retinal cells and (c) purified glial cells as they were ejected from the nozzle. The arrows indicate individual cells. Close-up images of (d) retinal cells and (e) glial cells. Scale bar: 100 μm. Reprinted with permission from [10^▪^].

A recent study performed by us has shown that adult rat retinal ganglion cells, which are neuronal cells of the central nervous system, as well as glial cells, another cell type in the retina, can be printed using piezo inkjet technology (Fig. 1) [10^▪^]. Piezo inkjet printing utilizes the rapid movements of a piezoelectric ceramic element to eject liquid drops from a nozzle. Although this technology is well adapted for industrial-scale printing because of its flexibility and reliability, it has been less commonly used to print cells as investigators were concerned that the vibration frequency in piezoelectric print heads may lead to cell membrane disruption and cause cell death [1,11].

It was, however, shown that the viability of the retinal cells was not significantly affected through the printing process, although a reduction of the cell number because of sedimentation within the print head did occur. Importantly, printed cells exhibited similar survival rates and regeneration (promoting) properties in culture compared with their nonprinted counterparts, suggesting that the cells are not adversely affected by the printing process [10^▪^]. Although this has been an exciting first step, the results of this study need to be extended to other cells of the retina to see if they can also be successfully printed. In addition, the printed retinal cells were deposited either as a monolayer in culture, or printed retinal ganglion cells were deposited on top of another printed retinal cell type, glial cells [10^▪^]. To move these findings forward toward creating a functional retina, they need to be translated into a complex three-dimensional cellular structure.

## CREATION OF CELLULAR STRUCTURES THROUGH THREE-DIMENSIONAL PRINTING

Several approaches have been taken to construct complex cellular structures over the past years using various printing techniques adapted to create three-dimensional structures.

These techniques include inkjet printing [7], laser-assisted printing [12], and micro-extrusion printing [5,6,13^▪▪^,^14^,15^▪^]. All these techniques have advantages and disadvantages. Inkjet printing allows greater deposition throughput, though it can be difficult to print fluids with high cell concentrations (>10 million cells/ml) because of issues with nozzle clogging and there is an increased risk of cell damage because of shear stress near the nozzles. Laser-assisted printing, the most expensive method, allows printing of concentrated fluids whilst maintaining cell viability, but is relatively slow and cell placement accuracy can be an issue. Finally, microextrusion printing is a relatively low-cost and speedy method that allows printing of highly concentrated cell suspensions. However, cell viability has been reported to be lower compared with inkjet printing [16].

One approach to construct cellular structures using these methods has been to print three-dimensional scaffolds using noncellular hydrogel. The scaffolds were subsequently coated with a growth-compliant substrate and seeded with hippocampal neuronal cells. Neurons were observed to thrive on these scaffolds and to form intricate networks [14].

In another approach, generally termed as bioprinting, crosslinkable matrix material and cells are deposited together in a defined pattern to construct a cellular three-dimensional structure. Initiated either photonic or thermally, the matrix materials can then be developed into scaffolds with tailored mechanical properties. Using this approach several complex biological structures have been created recently, including bone and cartilage-like constructs [7], heart [6], and skin tissue [5,12]. In addition, brain-like structures containing cortical neurons have recently been made [15^▪^].

For the creation of functional tissue which exhibits long-term cell survival another component, a functional vasculature, is critical. Creating multilayered tissue containing different cell types and a vascular network has been hindered by technical limitations so far. A recent study has described a novel three-dimensional bioprinting approach in which cells, extracellular matrix, and a synthetic vascular network were coprinted into a tissue construct. In this study, a custom designed three-dimensional bioprinter with four independently controlled printheads was used to sequentially coprint multiple inks, including cell-laden and fugitive inks, to create cellularized tissue that contains a vascular network [13^▪▪^].

## CHALLENGES OF CREATING A FUNCTIONAL RETINA THROUGH THREE-DIMENSIONAL PRINTING

**FIGURE 2:**
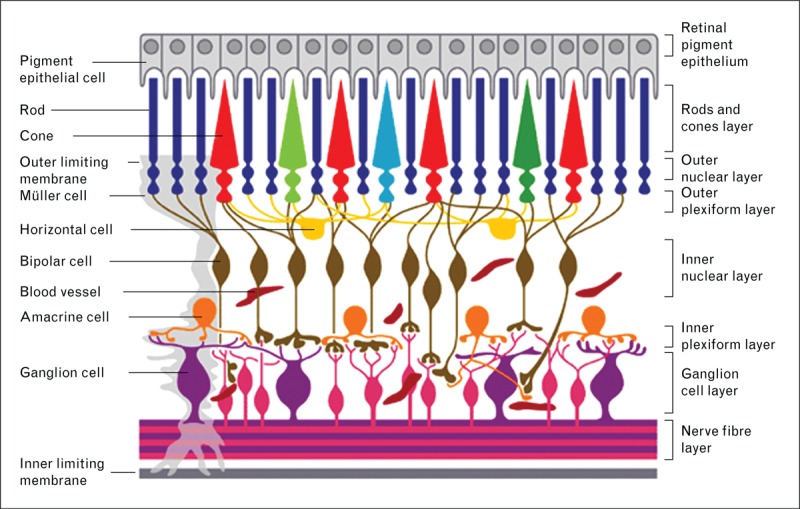
Cellular organization of the retina. Reprinted with permission from [18].

As described above, some promising steps have been taken toward printing a functional retina, although many more issues remain to be addressed. The human retina is a highly complex vascularized tissue that contains at least 60 functionally different cell types [17]. These can be broadly categorized into rod and cone photoreceptor cells, horizontal cells, bipolar cells, amacrine cells, retinal ganglion cells as well as support cells, glial cells, which include Müller cells (Fig. 2) [18].

These retinal cells need to act in concert with each other to successfully relay visual information from the eye to the brain. In certain retinal diseases only specific cells may need replacing, like the retinal ganglion cells in glaucoma, or the photoreceptor cells in retinitis pigmentosa, though other retinal cells may also have been affected through the primary cell loss [19]. In other circumstances certain areas of the retina may need replacement, as the macula in age-related macular degeneration. A printed macula could be surgically implanted into the eye. Whether it would successfully integrate remains to be seen. Finally, in advanced retinoblastoma or following severe ocular trauma the whole eyeball, including the retina, may need to be replaced. In support toward the latter, a noncellular, three-dimensional model of a human eye has recently been printed [20].

It has previously been shown that it is possible to successfully print rat retinal ganglion cells and glial cells using inkjet printing technology [10^▪^]. As a first step it needs to be investigated whether these findings can be translated to human retinal ganglion cells and glial cells and if human photoreceptor cells, horizontal, bipolar, and amacrine cells can also be successfully printed without loss of viability and function. In addition, three-dimensional printing technology needs to be employed to print the various cell types in specific spatial arrangements to mirror the highly complex retinal tissue structure. Although previous studies have shown that cellular structures containing a selective number of other, mostly nonneuronal, cell types can be created [4–8,12,13^▪▪^,^14^,15^▪^], the complexity needed to create functional organs and neuronal tissue like the retina has yet to be achieved.

The primary challenge includes printing cells accurately at high densities to achieve the high cell numbers needed that make up a functioning retina. This is perhaps most crucial in printing the rod and cone photoreceptor cells to make up the light-sensitive layer of the retina. To achieve the close-packed arrangement of the rod-cone receptor mosaic a biomimicry or self-assembly approach [16], in addition to accurate printing at the single cell level, will probably need to be employed. Similar approaches will be required to print other retinal cell types to allow them to synaptically connect together correctly and establish horizontal and vertical connections between cells in different layers to ensure proper physiological function and transmission of visual information in the printed retina. Furthermore, the retinal ganglion cells, a gateway to transmit visual information from the retina to the brain, need to retain their regeneration promoting properties to successfully extend their nerve processes through the optic nerve toward the brain. In this respect, it has been encouraging to see that printed retinal ganglion cells are able to successfully extend neurites in culture [10^▪^].

To maintain cell viability during the printing process, a reliable high-throughput printing method, such as industrial-scale multihead printing platforms, may need to be employed. As it was previously observed that cell sedimentation in the inkjet print head can lead to significant cell loss [10^▪^], modifications to reduce this phenomenon will need to be implemented to allow printing of cells over a prolonged period, without loss of yield [21,22]. With the continuous improvement of other three-dimensional printing methods, other printing processes, such as laser-assisted printing, may turn out to be better suited to achieve the goal of printing a functional retina. Additionally, to ensure long-term survival of the retinal cells by providing nutritional support and oxygen, ways need to be found to construct vascularised retinal tissue. As mentioned before, a recent study has provided an encouraging approach toward solving this issue [13^▪▪^]. It remains to be seen if this approach can be successfully applied to the retina.

Finally, any three-dimensional printed tissue, such as the retina, will need to undergo a rigorous approval process by the respective regulatory government body before it can be considered for human transplantation. The nature of an implantable, three-dimensional printed retina means that it will most likely be defined as a Class III medical device and therefore requires more stringent approval review processes such as premarket approval by the US Food and Drug Administration. As a result, the three-dimensional printing process will need to follow strict guidelines such as the US Food and Drug Administration's Current Good Manufacturing Practice. Although many Current Good Manufacturing Practice requirements, like aseptic condition and prevention of cross-contamination, are self-explanatory, their implementations are often nontrivial and potentially costly. For example, while industrial piezo print heads can offer high-throughput and excellent reliability, most cannot be properly sterilised because of the ways they are constructed. Furthermore, their internal fluid-contacting surfaces often consist of materials not proven to be inert to biological matter, so cross-contamination cannot be ruled out. Last but not least, validation of the functionality of a three-dimensional printed retina will need to be carried out before implantation into a patient. With several million retinal cells of different types working in concert to convert light input into neurological signals, developing an in-vitro analytical method to comprehensively verify the working of a three-dimensional printed retina can itself be a significant challenge.

## CONCLUSION

Recent advances in the construction of cellular three-dimensional structures have provided encouraging advances potentially relevant to the creation of a three-dimensional printed retina. In addition, it has recently been shown that certain mammalian retinal cells, adult rat retinal ganglion cells, and glia, can be successfully printed without loss of viability and certain phenotypic features. These findings now need to be translated to other cell types of the retina and to human tissue. Furthermore, to create a functional three-dimensional printed retina, challenges in the construction of complex three-dimensional printed tissue, including cell density, spatial, and functional integration of the various cell types and sustainment of long-term cell survival need to be addressed. Finally, realizing the potential of research to create an implantable three-dimensional printed retina still requires significant engineering development, regulatory effort, and ultimately, capital investment. Taken together, we believe that it is still a long way to go until a functional three-dimensional printed retina is on the horizon, but some promising results have shown that we are on the right track.

## Acknowledgements

None.

### Financial support and sponsorship

The research in K.R.M.'s laboratory was supported by a van Geest Fight for Sight Early Career Investigator Award, grant number 1868 (B.L.), the Cambridge Eye Trust (K.R.M.), the Jukes Glaucoma Research Fund (K.R.M.), the HB Allen Charitable Trust (K.R.M.) and core support grant from the Wellcome Trust and MRC to the Wellcome Trust – Medical Research Council Cambridge Stem Cell Institute (K.R.M.).

### Conflicts of interest

There are no conflicts of interest.

## REFERENCES AND RECOMMENDED READING

Papers of particular interest, published within the annual period of review, have been highlighted as:

▪ of special interest
▪▪ of outstanding interest
